# Assessing the dynamic performance of water companies through the lens of service quality

**DOI:** 10.1007/s11356-023-30779-z

**Published:** 2023-11-10

**Authors:** Ramon Sala-Garrido, Manuel Mocholi-Arce, Maria Molinos-Senante, Alexandros Maziotis

**Affiliations:** 1 Departament of Mathematics for Economics, University of Valencia, Avd. Tarongers S/N, Valencia, Spain; 2 National Research Center for Integrated Natural Disaster Management (CIGIDEN), CONICYT/FONDAP/15110017, Avda, Vicuña Mackenna, 4860 Santiago, Chile; 3 Institute of Sustainable Processes, University of Valladolid, C/ Mergelina S/N, Valladolid, Spain

**Keywords:** Cross-efficiency, Productivity, Undesirable outputs, Environmental variables, Water utilities, Data envelopment analysis

## Abstract

The measurement of performance within the water industry holds significant importance for policymakers, as it can help guide decision-making for future development and management initiatives. In this study, we apply data envelopment analysis (DEA) cross-efficiency techniques to evaluate the productivity change of the Chilean water industry during the years 2010–2018. Water leakage and unplanned interruptions are included in the analysis as quality of service variables. Moreover, we use cluster analysis and regression techniques to better understand what drives productivity change of water companies. The results indicate that the Chilean water industry is characterized by considerable high levels of inefficiency and low levels of productivity change. This is due to the existence of technical regress whereas gains in efficiency were small. Concessionary water companies were found to be more productive than full private and public water companies. Best and worst performers need to make efforts to reduce production costs and improve service quality. Other factors such as customer density and ownership type statistically affect productivity.

**Supplementary Information:**

The online version contains supplementary material available at 10.1007/s11356-023-30779-z.

## Introduction

The measurement of water industry performance plays a crucial role in defining its current situation and anticipating future conditions (Walker et al. 2021). The outcome of this analysis can help water regulators and regulated companies to design policies and incentives to guide development (Mergoni et al. 2022). The measurement of performance is linked to the evaluation of efficiency and productivity change where costs across water companies are compared for the purposes of setting tariffs (Nyathikala and Kulshrestha 2017). Efficiency provides a static evaluation of the performance of the units analyzed whereas the assessment of productivity change involves extending the notion of efficiency to an intertemporal setting (Lo Storto 2021). Moreover, productivity change consists of two components, catch-up and technical change. The former refers to the movement of less efficient units closer or away from the efficient frontier. The latter refers to the shift of the efficient frontier (or production possibilities frontier) due to technological innovation (Liu et al. 2020).

Over the years, there have been several studies in the water industry that have evaluated its performance (Berg and Marques 2011; Carvalho et al. 2012; See 2015; Pinto et al. 2017; Goh and See 2021; Pereira et al. 2023). While the majority of past research on this topic refers to developed countries, few studies exist for developing countries (Cetrulo et al. 2019). Our study aims to shed more light on this topic by evaluating the performance of water industry in Chile. From a regulatory perspective, the peculiarities of the water industry make Chile an interesting case study. First, the coverage of drinking water and wastewater treatment services in Chilean urban areas is almost universal. By contrast, in the other Latin American and Caribbean countries, on average, only 65% of the population has access to safely managed water services and 22% of them to sanitation services (PAHO 2018). Hence, Chile can be considered a paradigmatic country in terms of water and sanitation service in the context of Latin American and Caribbean countries. Second, the water sector followed its privatization process during the years 1998–2004 forming three types of companies (Molinos-Senante et al. 2018a). Full private water companies (FPWCs) which are the owners of the infrastructure and provide services to its customers for an infinite time period. By contrast, in the case of the concessionary water companies (CWCs) the infrastructure belongs to the state and water companies deliver water and wastewater services for a finite time period (i.e., 30 years) (Molinos-Senante et al. 2019). A small number of customers (less than 5% of the total national customers) receive drinking water from a public water company (PWC). Third, in order to protect customers from monopoly abuse, a water regulator, Superintendencia de Servicios Sanitarios, was set up to monitor financial and environmental performance and determine annual tariffs. The process to set water tariffs in Chile is based on the definition of a hypothetical economic efficient water company. Under this methodological approach, the maximum water tariff allows recovering the full costs of providing drinking water and wastewater treatment services assuming that the water company is efficient (Molinos-Senante et al. 2019).

There are two main frontier approaches to assess water industry performance. The first approach is the use of linear programming (non-parametric) methods such as data envelopment analysis (DEA), and the second approach is the use of econometric (parametric) such as stochastic frontier analysis (SFA). In the case of the Chilean water industry, the latter approach was employed by Molinos-Senante et al. (2018a) to evaluate the determinants of productivity change for full private and concessionary water companies in Chile. The results showed that productivity deteriorated due to technical regress whereas gains in efficiency were small. Although this technique incorporates both inefficiency and noise, its main limitation is the a priori assumption of a functional form for the underlying production technology. Other studies by Molinos-Senante and Sala-Garrido (2015) and Sala-Garrido et al. (2018, 2019) employed DEA techniques to assess productivity change. The studies corroborated previous findings by Molinos-Senante et al. (2018a) by reporting a retardation in industry productivity. Unlike SFA, DEA does not make any assumption for the functional form of the production technology (Suarez-Varela et al. 2017).

The previous studies used traditional DEA techniques to evaluate companies’ performance (Cetrulo et al. 2019; See 2015; Goh and See 2021). In conventional DEA models, the weight given to each variable is set to maximize the efficiency score of the unit evaluated. This method is termed “self-evaluation” because each water company’s efficiency is assessed using the most favorable weights specific to it (Liu et al. 2018). As a result, efficiency scores might be overestimated (Wang and Chin 2010). Moreover, some water companies could allocate a weight equal to zero for some variables used to estimate efficiency scores. This means that these variables are not considered in the efficiency assessment (Contreras 2020). To address this drawback, various methodological strategies have been suggested. One such strategy involves adding constraints to the weights assigned to inputs and outputs (Allen et al. 1997). This technique has predominantly been used to incorporate expert opinions in the efficiency evaluation process (Alberca and Santos 2021). On the other hand, Sexton et al. (1986) and Doyle and Green (1994, 1995) developed the concept of cross efficiency where the evaluation of each firm considers their own weights and the weights of the other firms evaluated, i.e., performance assessment is based on self and peer evaluation. However, the weights derived from this process might not be unique (Moeini et al. 2015), i.e., the same unit can have different weights, which limits the benchmarking process based on performance. To deal with this limitation, Doyle and Green (1994, 1995) developed additional linear programming models that could be solved to optimize the weights of the firms by treating them as collaborators (benevolent) or competitors (aggressive). Other studies (e.g., Wang and Chin 2010; Liu et al. 2018; Ding et al. 2019) employed a neutral formation as there is no certain criteria that ensures that firms are collaborators or competitors.

Cross-efficiency techniques are especially useful to benchmark the performance of water companies as they are heterogeneous entities that operate in areas with different environmental characteristics. To the best of authors’ knowledge, within the context of water industry, Sala-Garrido et al. (2021, 2023) stand as the only researchers who have assessed the performance of water companies using DEA cross-efficiency techniques. The case study developed by Sala-Garrido et al. (2021) focused on English and Welsh water companies and integrated greenhouse gas emissions as undesirable outputs. In contrast, Sala-Garrido et al. (2023) concentrated on Chilean water companies but did not incorporate undesirable outputs in their assessment. It is noteworthy that both of these prior studies centered on the evaluation of water companies’ efficiency, which involves appraising their performance at a specific point in time (Gémar et al. 2018). In distinction, this study takes a step further by delving into the concept of productivity change, which extends the notion of efficiency into a temporal context. Notably, no precedent of studies exists that have examined water companies’ productivity change based on cross-efficiency DEA techniques.

The main objective of our study is to evaluate the productivity change of the Chilean water industry using cross-efficiency methods. Because the current regulatory model applied in Chile to set water tariffs is based only on economic variables, we incorporate water leakage and unplanned water supply interruptions as undesirable outputs to get a more holistic assessment of the performance of water companies. To get a better insight on companies’ performance, we use cluster analysis to group companies into homogeneous groups based on their productivity scores. The final step of our assessment exercise uses regression techniques to discuss the role of operating characteristics, beyond companies’ control, on productivity change estimations. Consequently, this study seeks to answer two primary research questions: (i) over the period 2010–2018, did the productivity of Chilean water companies deteriorate or improve, and to what degree? and (ii) what were the internal and external factors, relative to the management practices of water companies, that influence the estimates of productivity change?

## Methodology

In this section, we outline the methodology used to calculate the efficiency and productivity change of the Chilean water industry using cross-efficiency DEA techniques. Let us assume that the industry consists of $$n$$ firms (water companies) and that each firm $$f \left(f=1,\dots ,n\right)$$ uses a vector of inputs $${x}_{if}$$ ($$i=1,..,m)$$ to generate a vector of desirable outputs denoted as $${y}_{lf} \left(l=1,\dots ,k\right)$$ and a vector of undesirable outputs denoted as $${b}_{sf} \left(s=1,\dots ,r\right)$$. In this study, we are interested in analyzing the performance of water companies incorporating undesirable outputs such as water leakage and unplanned interruptions.

The negative undesirable outputs were converted to positive ones by applying the following transformation equation (Seiford and Zhu 2002; Liu et al. 2017; Ding et al. 2019):

1 $$\widehat{{b}_{s}}=-{b}_{s}+{w}_{s}>0, (s=1, 2, ..., r)$$

where ${w}_{s}$ is a proper $n-$ dimensional vector. Given that all translated outputs are positive, the constant ${w}_{s}$ needs to be a value that is greater than or, equal to the highest observed value for each undesirable output indicator $b$ across all units (Zanella et al. 2015).

Thus, the efficiency of firm $f$ with respect to itself, ${\varphi }_{ff}$ is obtained by solving the following linear programming:

2 $$Max\;{\varphi }_{ff}=\sum \nolimits_{l=1}^{k}{\mu }_{lf}{y}_{lf}+\sum \nolimits_{s=1}^{r}{\omega }_{sf}\widehat{{b}_{sf}}$$

$$\sum \nolimits_{i=1}^{m}{v}_{if}{x}_{if}=1$$

$$\sum \nolimits_{i=1}^{m}{v}_{if}{x}_{ij}-\sum \nolimits_{l=1}^{k}{\mu }_{lf}{y}_{lj}-\sum \nolimits_{s=1}^{r}{\omega }_{sf}\widehat{{b}_{sj}}\ge 0\,j=1,\dots ,n$$

$${v}_{if}\ge 0, {\mu }_{lf}\ge 0,{\omega }_{sf}\ge 0$$

where ${\mu }_{lf}, {\omega }_{sf}, \mathrm{and} {v}_{if}$ present the weights for each desirable output, undesirable output and inputs, respectively. The linear programming model (2) is the traditional DEA model introduced by Charnes et al. (1978) which presents the limitations previously discussed. To overcome these limitations, in the next stage of assessment, we follow Wang and Chin (2010) and Ding et al. (2019) approaches and solve a neutral linear programming model^1^ where the efficiency of each firm is determined using weights only from its own point of view without taking into account their effects on other firms (Wang and Chin 2010). This linear programming model is defined as follows:

3 $$Max \,\beta$$

$$\sum \nolimits_{i=1}^{m}{v}_{if}{x}_{if}=1$$

$$\begin{array}{cc}\sum_{i=1}^{m}{v}_{if}{x}_{ij}-\sum_{l=1}^{k}{\mu }_{lf}{y}_{lj}-\sum_{s=1}^{r}{\omega }_{sf}\widehat{{b}_{sj}}\ge 0 & j=1,\dots ,n, j\ne f\end{array}$$

$$\sum \nolimits_{l=1}^{k}\begin{array}{c}{\mu }_{lf}{y}_{lf}+\sum_{s=1}^{r}{\omega }_{sf}\widehat{{b}_{sf}}-{\varphi }_{ff}\sum_{i=1}^{m}{v}_{if}{x}_{if}=0\\ \end{array}$$

$$\begin{array}{cc}{\mu }_{lf}{y}_{lf}-\beta \ge 0& l=\mathrm{1,2},\dots . k\end{array}$$

$$\begin{array}{cc}{\omega }_{sf}{b}_{sf}-\beta \ge 0& s=\mathrm{1,2},\dots . r\end{array}$$

$${v}_{if}\ge 0, {\mu }_{lf}\ge 0,{\omega }_{sf}\ge 0,\beta \ge 0$$

where ${\varphi }_{ff}$ is the efficiency of firm $f$ relative to itself which was derived solving model (2). After obtaining the optimal weights ${\mu }_{lf}^{*}, {\omega }_{sf}^{*},{v}_{if}^{*}$, we calculate the cross-efficiency of each firm $j$ corresponding to firm $f$ as follows (Ding et al. 2019):

4 $${CE}_{fj}=\frac{\sum_{l}^{k}{\mu }_{lf}^{*}{y}_{lj}+\sum_{s=1}^{r}{\omega }_{sf}^{*}\widehat{{b}_{sj}}}{\sum_{i=1}^{m}{v}_{if}^{*}{x}_{ij}}$$

Therefore, the cross-efficiency of each firm $j$ is derived as follows:

5 $${CE}_{j}=\frac{1}{n}\sum \nolimits_{f=1}^{n}{CE}_{fj}$$

The linear programming model (3) can be used as well to assess the productivity change of each firm over time. In particular, we use the traditional Malmquist productivity index (MPI) (Fare et al. 1994) and estimate it with cross efficiency techniques. The cross-efficiency MPI ($CE\_MPI$) between time periods $t$ and $t+1$ is as follows (Ding et al. 2019):

6 $$CE\_MPI={\left(\frac{{CE}^{t+1}\left({x}^{t+1},{y}^{t+1},{b}^{t+1}\right)}{{CE}^{t+1}\left({x}^{t},{y}^{t},{b}^{t}\right)}\times \frac{{CE}^{t}\left({x}^{t+1},{y}^{t+1},{b}^{t+1}\right)}{{CE}^{t}\left({x}^{t},{y}^{t},{b}^{t}\right)}\right)}^\frac{1}{2}$$

A $CE\_MPI$ greater than 1 suggests an improvement in productivity whereas a value less than 1 implies a deterioration in productivity. The cross-efficiency MPI can be further decomposed into two components, cross-efficiency change (CEC) and cross-efficiency technical change (CETC). This decomposition is provided below:

7 $$CE\_MPI=\frac{{CE}^{t+1}\left({x}^{t+1},{y}^{t+1},{b}^{t+1}\right)}{{CE}^{t}\left({x}^{t},{y}^{t},{b}^{t}\right)} \times {\left(\frac{{CE}^{t}\left({x}^{t+1},{y}^{t+1},{b}^{t+1}\right)}{{CE}^{t+1}\left({x}^{t+1},{y}^{t+1},{b}^{t+1}\right)}\times \frac{{CE}^{t}\left({x}^{t},{y}^{t},{b}^{t}\right)}{{CE}^{t+1}\left({x}^{t},{y}^{t},{b}^{t}\right)}\right)}^\frac{1}{2}=CEC \times CETC$$

The efficiency terms, ${CE}^{t}\left({x}^{t},{y}^{t},{b}^{t}\right)$ and ${CE}^{t+1}\left({x}^{t+1},{y}^{t+1},{b}^{t+1}\right)$ are solved using the linear programming model (3). The efficiency scores that use mixed period technology and data, ${CE}^{t}\left({x}^{t+1},{y}^{t+1},{b}^{t+1}\right)$ and ${CE}^{t+1}\left({x}^{t},{y}^{t},{b}^{t}\right)$ are also solved based on the linear programming model (3) (for more details, please see Appendix). Their related cross-efficiency scores are then derived using Eqs. (4) and (5). In Eq. (7), efficiency change measures how less efficient companies improved their efficiency over time towards the most efficient ones (catch-up). Technical change captures the shift to the efficient frontier due to advances in technology. If efficiency change takes a value greater (lower) than one, then the firm achieved gains (losses) in efficiency. If technical change takes a value greater (lower) than one, then the firm experienced technical progress (regress). Both components can therefore contribute to change in productivity positively or negatively.

To examine the potential grouping of evaluated water companies based on their productivity change scores, we employ cluster analysis techniques. In particular, each yearly productivity change score for the water companies, i.e., $CE\_MPI$ estimations on Eq. (6) are employed as the variable for clustering. With this approach we can identify best and worst groups of water companies in terms of productivity and use this information to design group specific policies to enhance performance. Cluster analysis is a statistical technique where a dataset is grouped into a set of clusters such that data that go with the same cluster are similar and data from different clusters are dissimilar (Velmurugan 2018). There were several studies in the past that used cluster analysis to group companies based on their performance scores derived from linear programming techniques (for a review, see Samoilenko and Osei-Bryson 2010; Jiang et al. 2019). Other studies highlighted the advantage of linking DEA and cluster analysis in improving decision making process (Lemos et al. 2005; Bojnec and Latruffe 2007; Sikka et al. 2009; Omrani et al. 2018; Cinaroglou 2019).

Numerous clustering algorithms have been proposed and studied in the literature (Velmurugan 2018). While each method has its own advantages and disadvantages, our study opts for the k-medoid technique due to its favorable characteristics that align well with our specific case study (Velmurugan 2018; Firsova and Chernyshova 2020). K-medoid clustering technique is a similar to K-means. It partitions the data in $k$ clusters by attempting to minimize the distance of the data points belonging to a cluster from the center of the cluster. In contrast to k-means algorithm, the centre of the cluster is an existing data entry (medoid) and not the average points of the cluster. This allows for better interpretability of the clustering results. K-medoids algorithm minimizes the sum of pairwise dissimilarities, instead of the sum of square Euclidean distances that K-means uses.

The K-medoid algorithm works as follows. The first step is the initialization step which involves a greedy process to select the k-medoids by computing the changing in the dissimilarity criterion for each new medoid. In the second step, each data-point is clustered with its closest medoid, where the closest medoid is the medoid with smaller dissimilarity measure. After selecting the $k$ clusters, the medoid of each cluster should be updated. This is because the partitioning process was based on a greedy process and not an extensive search. Thus, the third step is an iterative process which updates the medoids of the clusters. In order to choose the optimal number of clusters $k$, we use the silhouette value (Cinaroglou 2019), which gives an indication of the similarity among the data points that are in the same cluster. The higher the value of the silhouette score, the higher the similarity (Tan et al. 2005). Thus, we select the number of clusters which have the highest silhouette score.

The last step of our analysis explores whether other operating characteristics beyond water companies control such as customer density and ownership could impact their performance. The conditional DEA methodology, proposed by Daraio and Simar (2005), provides an excellent way to account for environmental factors when assessing efficiency. However, the partial frontiers created by these conditional DEA models rely on a self-assessment approach. Given that this research determines productivity change based on both self- and peer-evaluation, we have chosen regression analysis as the technique to pinpoint the external variables that impact the performance of water companies. In doing so, we use the estimates from the cross efficiency MPI as the dependent variable and regress them against a set of operating characteristics (Ananda 2019). The regression model is defined as follows (Guerrini et al. 2015; Zhang et al. 2016; Wang et al. 2017):

8 $${CE\_MPI}_{j,t}={\gamma }_{0}+{\gamma }_{j}{\pi }_{j,t}^{\prime}+{\tau }_{j}+ {\varepsilon }_{j,t}$$

where ${CE\_MPI}_{j,t}$ is the cross efficiency MPI score obtained from Eq. (7), ${\gamma }_{0}$ denotes the constant term, ${\pi }_{j,t}^{\prime}$ is the vector of operating characteristics of each water company at any time $t$, ${\tau }_{j}$ captures unobserved heterogeneity which is not uncorrelated with explanatory variables and ${\varepsilon }_{j,t}$ is the noise which follows the normal distribution. Finally, in the regression model we also included dummies for each company and year considered in the study.

## Empirical framework and data

The empirical application conducted focused on the Chilean water industry because, as we have discussed in the introduction, it presents several peculiarities from the regulatory perspective. The data in this study was collected from the website of the Chilean water regulator and covers the years 2010–18. Our sample consists of 11 full FPWCs, 9 CWCs, and 1 PWC. Whereas the water industry involves 54 water companies, the 21 analyzed in this study provide drinking water and wastewater treatment services to more than 90% of the urban customers (SISS 2022). Moreover, the 21 water companies are distributed across the whole country.

Over the past 25 years, there has been a notable surge in the volume of literature dedicated to benchmarking the water industry (Goh and See 2021). In contrast to earlier periods, where studies primarily centered on the English and Welsh water industry due to data accessibility through public channels, recent years have witnessed an expansion of performance assessments to encompass a multitude of countries (See 2015). This broader scope has led to a diversification of the variables, both inputs and outputs, used for evaluating the performance of water companies. As highlighted by See (2015), more than 50 different variables have been utilized in at least one study as either inputs or outputs. Given this vast array of potential variables available for efficiency assessment, our study has focused on those most commonly employed in past research specific to Chilean water companies (Molinos-Senante et al. 2019; Cetrulo et al. 2019; Goh and See 2021; Sala-Garrido et al. 2019; Maziotis et al. 2020).

Two desirable outputs were used. The first output was the volume of water delivered measured in thousands of cubic meters per year. The second output was the number of customers receiving wastewater treatment.^2^ As the Chilean water industry has carried programs to improve both the quality of the drinking water and wastewater treated, we adjusted both outputs to reflect these quality changes. In doing so, the volume of water delivered and the number of wastewater treatment customers were multiplied with the drinking water quality index and the wastewater treatment quality index, reported by the water regulator, respectively^3^ (Molinos-Senante and Sala-Garrido 2015; Sala-Garrido et al. 2019). We employed two undesirable outputs. The first was the volume of water leakage measured in thousands of cubic meters per year. It should be noted that the difference between the volume of water delivered and the volume of water leakage is not the volume of water billed because water companies also present apparent losses (Alegre et al. 2000). The second undesirable output was defined as the number of water supply unplanned interruptions measured in hours per year. Water companies also experience planned water supply interruptions and therefore, the difference between the “total time” and “unplanned water supply interruptions” does not directly equate to the duration of “water supply availability.” For the conversion of undesirable outputs, according to Eq. (1), the vector utilized consisted of the following values: (water leakage, water supply unplanned interruptions) = (16,717,000 m^3^/year, 141,435 h/year). For other case studies, alternative quality of service variables could be integrated in the assessment based on the main features of the water industry analyzed.

Two inputs were used. The first input was defined as the operating expenditure to provide both drinking water and wastewater treatment services to customers expressed in thousands CLP per year. The second input was defined as sum of the length of drinking water and wastewater networks measured in kilometers (km).

The choice of operating characteristics, often referred to as environmental variables, which could potentially influence the productivity of water companies, was guided by a comprehensive literature review conducted by Goh and See (2021). Following an exhaustive examination of 142 articles on benchmarking the performance of the water and sewerage industry across 26 different countries, Goh and See (2021) identified the most commonly considered environmental variables. These included the ownership structure of water companies, the source of water, levels of non-revenue water, and customer density. Consequently, based on Goh and See (2021), three specific environmental variables were selected in our analysis.

The first one was the customer density which was defined as the ratio of number of customers to the drinking water network length. The second environmental variable was the source of the raw water which is a categorical variable and captures surface, groundwater, and mixed water resources. The third environmental variable was the type of ownership: (i) FPWC, (ii) CWC, and (iii) PWC. These variables represent the operating characteristics vector for each water company at any given moment $t$ (${\pi }_{j,t}^{\prime}$) in Eq. (8).

Tables 1 and 2 depict a summary of the descriptive statistics for the variables used in the study.

**Table 1:** Descriptive statistics of the variables to assess performance of Chilean water companies. Average values for 2010–2018 period

Year	Volume of water delivered (000 m^3^/year)	Customers receiving wastewater treatment (nr)	Operating expenditure (000 s CLP/year)	Network length (km)	Drinking water quality indicator (Index)	Wastewater treatment quality indicator (Index)	Volumes of water leakage (000 m^3^/year)	Water supply unplanned interruptions (h/year)
2010	31,100,666	3254	45,703	0.978	687,311	0.974	13,689	103,686
2011	32,624,218	3265	47,518	0.971	698,236	0.954	13,685	75,278
2012	33,513,404	3301	48,594	0.973	686,474	0.957	14,023	104,005
2013	37,031,073	3367	49,451	0.974	704,605	0.962	14,270	141,432
2014	39,407,932	3403	50,418	0.972	722,549	0.988	14,280	121,834
2015	38,026,105	3421	51,185	0.986	740,264	0.973	14,137	109,744
2016	40,665,719	3478	52,166	0.988	754,255	0.975	14,905	33,540
2017	40,848,731	3587	52,892	0.987	769,340	0.971	15,112	33,271
2018	42,132,064	3687	54,473	0.981	791,968	0.950	15,564	29,286

Observations: 189

Costs are expressed in 2018 prices

**Table 2:** Number of water companies and its percentage for the categorical exogenous variables

Ownership	Full private	Concessionary	Public
	11 (52.4%)	9 (42.9%)	1 (4.7%)
Source of raw water	Groundwater	Surface	Mixed
	7 (33.3%)	2 (9.6%)	12 (57.1%)

## Results and discussion

**Fig. 1:**
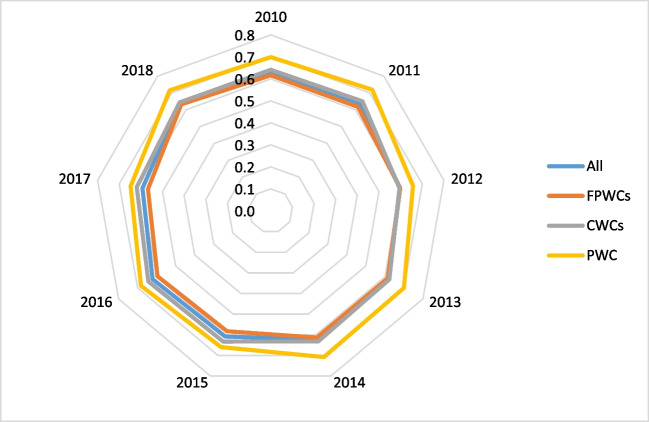
Average cross-efficiency scores for Chilean water companies according to its ownership: full private (FPWCs), concessionary (CWCs), and public water companies (PWC)

Figure 1 shows the evolution of the average cross efficiency scores during the years 2010–2018 by ownership type. It is found that during the period of study, the Chilean water industry was 0.618 efficient on average. This means that the water companies could reduce their costs and improve quality of service by 38.2% on average. This result is consistent with a previous study by Molinos-Senante et al. (2018b) who found that the Chilean water sector could further reduce its average costs by 39% to generate the same level of output.

Focusing on the ownership of the water companies, our results further indicate that the PWC was more efficient than private water companies with concessionary companies being more efficient than full private ones. It should be noted that in Chile, there is only one PWC, and therefore, conclusions should be interpreted with caution avoiding the generalization of the results. Nevertheless, it is found that on average the potential saving in costs and improving quality of service for the PWC could reach the level of 31.3%. Higher reductions should be achieved by full private and concessionary companies, 39.6% and 36.9% on average, respectively.

Overall, the results from the efficiency scores revealed two main points. First, the efficiency of the Chilean water industry is low. Regardless of ownership, water companies need to considerably reduce their production costs and improve the quality of service such as water leakage and the frequency of unplanned water supply interruptions. Second, efficiency scores are almost constant across years which illustrates that those measures for improving efficiency have not been satisfactory implemented by water company. Considering that water tariffs in Chile are set based on the definition of an “efficient” water company, it is evidenced that the regulator needs to introduce modifications to this regulatory model as water companies are far from being efficient.

Table 3 presents the main statistics of the productivity change of Chilean water companies from 2010 to 2018. It is concluded that on average the productivity of the water industry declined by 0.111% which was mainly attributed to a small increase in efficiency change of 0.527% and a small decrease in technical change of 0.497%. This finding implies that less efficient companies made some improvements in their efficiency towards the most efficient ones. However, the lack of advancing in new technologies did not allow them to improve productivity over time. It should be noted that productivity change estimations embrace two quality of service indicators, i.e., water leakage and unplanned water supply interruptions.

**Table 3:** Descriptive statistics of cross-efficiency Malmquist productivity index (CE_MPI) and its drivers (cross-efficiency change, CEC; cross-efficiency technical change, CETC) for Chilean water companies from 2010 to 2018

	Average	Minimum	Maximum	Standard deviation
CEC	1.005	0.949	1.078	0.043
CETC	0.995	0.934	1.058	0.039
CE_MPI	0.999	0.981	1.026	0.015

**Fig. 2:**
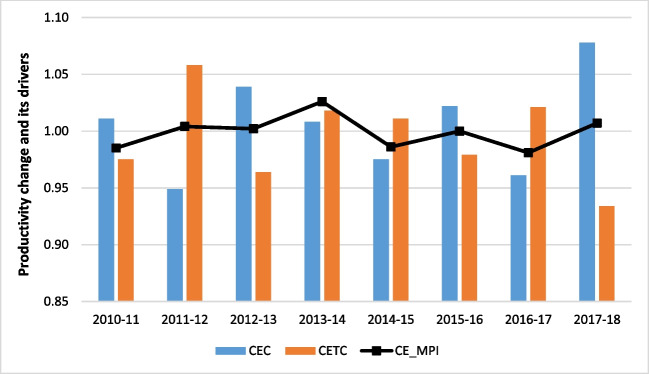
Cross-efficiency Malmquist productivity index (CE_MPI) and its drivers (cross-efficiency change, CEC; cross-efficiency technical change, CETC) for Chilean water companies

Detailed results of productivity change across years are shown in Fig. 2. It reflects the influence of quality of service variables on the performance of the Chilean water companies. Hence, according to the management reports published yearly by the Chilean water regulator (SISS 2022), the annual volume of water lost, for the water companies evaluated, in 2010 was around 344 million of cubic meters whereas this figure increased up to 384 million on 2018. In the case of unplanned water supply interruptions, a better performance was achieved in 2018 when the water outage was estimated at 29,286 h/year while in 2010 was 103,686 h/year. Nevertheless, it should be noted that on 2010, a moment magnitude M_w_ = 8.8 earthquake struck the west coast of the Maule region in Chile (Alberto et al. 2022). According to WHO (2010), 114 urban systems suffered damage nationally, of which 49 systems were considered to undergo severe damage. In the Chilean regions of Maule and Bio Bío, the drinking water suffered interruptions. Hence, in terms of unplanned water supply interruptions, 2010 could be considered as an atypical year due to the extreme natural hazard impacted the drinking water supply system.

Past research (Sala-Garrido et al. 2018; 2019) has also identified extreme natural events as possible causes of the negative productivity change of water companies in Chile. For example, Sala-Garrido et al. (2019) identified that the several extreme hydro meteorological events occurred in Chile in 2015 resulted in unplanned interruptions in the water and sewerage network leading to higher operating costs influencing negatively on the productivity change of water companies. Turning our attention to the other variables, i.e., desirable outputs and inputs, taken into account in productivity change estimations, Table 1 reveals that variations over the years are relatively restrained and exhibit less volatility compared to those related to unplanned water supply interruptions. This underscores the importance of incorporating service quality variables when evaluating the performance of water companies.

Results from this study are consistent with previous studies by Molinos-Senante and Sala-Garrido (2015) and Molinos-Senante et al. (2018a) who reported a retardation in water industry productivity with technical change being the factor that contributed negatively to productivity change. During the years 2011–2014, productivity followed an upward trend which was attributed to both efficiency change and technical change. It increased from 0.985 during the years 2010–2011 to 1.026 during the years 2013–2014, showing therefore an increase at a small rate of 0.433% on average. Efficiency change remained positive except for the years 2011–2012 which declined by 5.126%. During the years 2011–2014, it was increasing at an annual rate of 0.169% on average. This means that although less-efficient companies caught up with the most efficient ones, gains in efficiency were not considerable. This is mainly explained by the fact that during the years 2011–2014 operating costs and unplanned interruptions were increasing at 5.7% and 11.4% per year, respectively. Moreover, technical change was volatile but showed small increases in its rate by 0.377% per year on average. The year 2013–2014 was the only period in our sample where both efficiency change and technical change showed positive rates, 0.789% and 1.818% on average. The upward trend in productivity did not continue the following years. Any gains in efficiency were offset by technical regress contributing therefore negatively to productivity changes. Efficiency was increasing at a rate of 0.884% per year on average whereas technical change decreased at a rate of 1.372% per year on average. Although unplanned interruptions appeared to go down over time, water leakage continued to rise at a rate of 1.7% per year on average. We note that in 2018 the average level of water leakage remained at high levels, 32.3%.

Table 4 displays the decomposition of the cross efficiency MPI by ownership type. The results show several interesting conclusions. First, CWCs were more productive than FPWCs and the PWC. During the period of study, concessionary companies’ productivity improved whereas full private and public companies’ productivity deteriorated. The major determinant of productivity change for CWCs was efficiency change. Technical change was the driver who had the most adverse impact on productivity change for full private and public water companies. Our results showed that the productivity of FPWCs reduced by 0.701% which was attributed to a small increase in efficiency change by 0.604%. In contrast, technical change decreased at a rate of 0.962% on average contributing therefore negatively to productivity. Higher rates of technical regress were found for the PWC, − 2.741% on average. Thus, PWC productivity decreased by 2.515% on average. As for CWCs gains in efficiency and technical progress contributed positively to productivity, which increased by 0.461% on average.

**Table 4:** Cross-efficiency Malmquist productivity decomposition by ownership for Chilean water companies

	2010–2011	2011–2012	2012–2013	2013–2014	2014–2015	2015–2016	2016–2017	2017–2018	Average
Full private									
CEC	1.001	0.974	1.033	1.003	0.950	1.022	0.954	1.111	1.006
CETC	0.973	1.031	0.967	1.020	1.035	0.971	1.015	0.912	0.990
CE_MPI	0.973	1.003	0.997	1.019	0.980	0.994	0.969	1.010	0.993
Concessionary									
CEC	1.021	0.922	1.044	1.013	1.011	1.019	0.970	1.034	1.004
CETC	0.983	1.096	0.965	1.014	0.987	0.994	1.025	0.962	1.003
CE_MPI	1.004	1.008	1.008	1.026	0.997	1.012	0.992	0.992	1.005
Public									
CEC	1.028	0.913	1.066	1.011	0.932	1.034	0.948	1.105	1.005
CETC	0.928	1.026	0.915	1.038	0.974	0.928	1.041	0.931	0.973
CE_MPI	0.954	0.937	0.976	1.049	0.908	0.960	0.987	1.029	0.975

Our findings are consistent with a previous study by Sala-Garrido et al. (2019) who found that during the years 2010–2016, average productivity of FPWCs reduced by 7.5% whereas CWCs´ productivity improved by 0.5% on average. Technical change was the driver who contributed negatively to productivity. Full private companies’ productivity followed an upward trend during the years 2010–2014 mainly due to gains in efficiency suggesting that less efficient companies made some improvements in their managerial practices. However, these gains were offset by the inability of the firms to adopt best industry’s practices. Technical regress was also apparent during the years 2015–2018 with a rate of 1.7% on average per year. Thus, FPWCs need to considerably be more technologically innovative to improve productivity. Similar conclusions are drawn for the PWC. Technical regress was the dominant driver of productivity change. During the years 2011–2014, technical change was volatile and fell by 2.33% per year on average, whereas efficiency change remained positive. In the following years efficiency change continued to increase by 0.48% per year on average. However, the lack of technological leadership led to a decrease in average productivity by 3.15% per year. Like full private companies, the PWC needs to make considerable investments in new technologies that could allow them to improve performance.

The regression on the productivity of the PWC is mainly attributed to an extreme deterioration in its quality of service. Thus, from 2010 to 2018, the variations in desirable outputs and inputs were relatively subdued. The operation costs for the PWC witnessed an upsurge of 11.0% between these years, while the quality-adjusted volume of water delivery experienced a growth of 9.7%. Conversely, there was a decline of 9.9% in the quality-adjusted number of people having access to wastewater during this period. By contrast, the percentage of non-revenue water in 2018 was 64.8% which involves that around of 48.6% of the abstracted water is lost in the production or distribution systems (SISS 2018). The performance of the PWC in terms of non-revenue water has been getting worse over time since in 2010 it has 42.9% which means that water lost was 32.2% (SISS 2010). It is surprising and disturbing the large percentage of water lost reported across years by the Chilean PWC because it is well known that water leakage is considered an example of economic, social, and environmental inefficiency in the water supply process (Molinos-Senante et al. 2016). In the case of the Chilean water industry, this poor performance, in terms of water lost, is even more relevant because according to the regulatory model used to set water tariffs, the maximum percentage of water leakage of an “efficient” water company is 15% (Molinos-Senante et al. 2019). This implies that if water losses exceed 15%, the financial burden should fall on the water company, as it cannot be included in the water tariff. However, data suggests that PWC is passing on the cost of lost water to consumers, given that over time, its water losses have not decreased but have in fact have risen. This finding is very relevant from a regulatory perspective since it evidences one of the main problems of the regulation of natural monopolies, i.e., the asymmetry of information (Bustos and Galetovic 2002).

As productivity scores showed considerable variation across water companies and over time (see Appendix), we used cluster analysis to group companies into homogeneous groups based on their cross efficiency MPI values over the whole period. The optimal number of clusters was selected based on the highest value of the silhouette measure (Tan et al. 2005). Therefore, two clusters were identified (see Appendix for more details); the first cluster is defined as the worst productivity group, whereas the second cluster denotes the most productivity group.

Table 5 contains a brief description of the clustering analysis results. Over the whole period, the worst performing group includes 12 water companies whose productivity score was 0.981 on average. This means that for this group of water companies, productivity deteriorated by 1.87% on average. Within this group the rates of productivity retardation varied between 0.30% and 5.85%. In contrast, the most productive group includes 9 water companies which had an average productivity score of 1.018. On average, this group of water companies improved their mean productivity by 1.8% which was considerably higher than the worst performing group. The worst performing group is characterized by water companies who have high operational costs and long networks to maintain because they have more customers to deliver water. In addition, this group of companies is described by high volume of water leakage but low levels of unplanned interruptions compared to the most productive group. In contrast, the most productive group is characterized by lower production costs and a smaller number of customers to provide services. Within this group of water companies, the volume of water leakage is lower but unplanned water supply interruptions are higher.

**Table 5:** Cluster analysis of Chilean water companies according to average cross-efficiency Malmquist productivity index values (2010–2018)

	Average	Minimum	Maximum	Water companies
Cluster I	0.981	0.942	0.997	FPWC1, FPWC2, FPWC5, CWC6, CWC7, PWC8, FPWC14, FPWC17, FPWC18, FPWC19, FPWC20, FPWC21
Cluster II	1.018	0.999	1.061	FPWC3, CWC4, CWC9, CWC10, FPWC11, CWC12, CWC13, CWC15, FPWC16

We note that the percentage of water leakage is similar for both groups of water companies which means that any differences in the volume of water leakage are due to the higher volume of drinking water supplied which reflect differences in their customer base. Therefore, the subpar performance of certain water companies cannot be attributed to their service quality. Instead, it relates to the other variables (inputs and desirable outputs) used in calculating productivity change (CE_MPI). However, they need to improve their daily operations. For instance, they could invest in technologies (e.g., less-energy intensive) that could help them reduce the cost of abstraction, treatment and distribution of water and thus, overall production costs. A second implication of this finding is that scale of operations might require adjustments. For instance, smaller water companies might be justified in terms of costs savings. In contrast, the most productive group could further improve productivity by improving the quality of service such as the level of water leakage and unplanned interruptions. Moreover, there might be other operating characteristics that could impact companies’ productivity. The worst productivity group takes water from mixed water resources, both surface and groundwater resources. The most productive group is characterized by large customer density which means that it is less costly to connect pipes and deliver water to a lower number of people. In order to better understand the impact of these operating characteristics that are beyond companies’ control, we proceed to the final step of our analysis (see Table 6).

**Table 6:** Influence of operational characteristics on the productivity change of Chilean water companies. Estimates of regression

Variables	Coeff	St.Err	*Z*-stat	*p* value
Constant	0.917	0.044	**20.700**	0.000
Customer density	0.002	0.001	***1.920***	0.055
Type of water resource	0.099	0.066	1.490	0.135
Type of ownership	0.072	0.029	**2.520**	0.012
Year				
2012	0.066	0.013	**5.076**	0.000
2013	0.057	0.013	**4.384**	0.000
2014	0.043	0.013	**3.320**	0.001
2015	0.005	0.013	0.370	0.712
2016	0.029	0.015	**1.970**	0.049
2017	0.006	0.014	0.440	0.656
2018	0.029	0.014	**2.080**	0.038
Water company (WC)				
WC1	− 0.138	0.035	** − 3.942**	0.000
WC2	0.111	0.024	**4.625**	0.000
WC3	0.114	0.036	**3.220**	0.001
WC4	− 0.145	0.040	** − 3.625**	0.000
WC5	0.141	0.037	**3.810**	0.000
WC6	0.145	0.035	**4.142**	0.000
WC7	− 0.054	0.064	− 0.850	0.396
WC8	0.101	0.051	**1.970**	0.049
WC9	0.182	0.077	**2.370**	0.018
WC010	0.013	0.024	0.520	0.603
WC11	0.145	0.060	**2.430**	0.015
WC12	0.179	0.073	**2.450**	0.014
WC13	0.075	0.036	**2.120**	0.034
WC14	0.177	0.072	**2.450**	0.014
WC15	0.127	0.021	**6.070**	0.000
WC16	− 0.055	0.043	− 1.270	0.205
WC17	0.017	0.028	0.590	0.557
WC18	− 0.117	0.073	− 1.600	0.110
WC19	0.141	0.031	4.548	0.000
WC20	0.131	0.026	5.038	0.000
Wald X^2^	**89.690**			
*p* value	0.000			

CE_MPI estimation is the dependent variable. Bold statistics are statistically significant at 5% significance level. Bold and italic statistics are statistically significant at 10% significance level

The results on Table 6 indicate that customer density and type of ownership had a statistically significant impact on water companies’ productivity. In particular, it was found that keeping other variables constant, a 1% increase in customer density might result in a 0.002 unit increase in company’s productivity on average. The existence of economies of density might explain the low levels of efficiency and productivity reported in the Chilean water industry since with the exception of Santiago, the capital city of the country, the density of customers is moderate for the other Chilean cities. The positive and statistically significant parameter regarding the type of ownership demonstrates from a statistical perspective that concessionary companies were more productive than full private and public water companies.

## Conclusions

The identification of best and worst performers is crucial for regulated water industries in crafting incentives and strategies to enhance performance. Accurate methods are essential for computing performance scores, as unreliable results from the benchmarking process will not be accepted by the evaluated water companies. To address this, our study breaks new ground by evaluating the productivity change of a selection of water companies using cross-efficiency methods, and incorporating the lack-of-service quality variables as undesirable outputs.

The case study focused on the Chilean water industry, a paradigmatic case within the Latin American and Caribbean regions. This is because urban areas in Chile boast near-universal coverage of drinking water and wastewater treatment services. Cross-efficiency findings revealed that Chilean water companies were generally inefficient, with an average potential savings in costs and undesirable outputs standing at 38.2% for generating the same output level. Productivity change assessments showcased a slight decline in the industry’s productivity over the years. Minor efficiency boosts of 0.527% had a positive influence on productivity, but this was offset by a technical regress of 0.497%, which negatively impacted it. In comparison, CWCs seemed more productive than both FPWCs and the PWC. This observation was statistically validated in the subsequent phase of the analysis.

Despite the novelty of the performance methodology applied and the findings presented in this study, it is important to acknowledge certain limitations. Firstly, our consideration of quality of service variables was confined to water supply interruptions and water leakage. However, on the global scale, there is a growing emphasis on other variables related to the environmental sustainability of water companies, such as carbon footprint, energy efficiency, and reagent consumption. Future research endeavors may explore the performance of Chilean municipalities by integrating additional quality of service variables with a particular focus on environmental performance. Secondly, the scope of our investigation into potential variables influencing the productivity change of water companies was also somewhat restricted. Previous research has indicated that there are additional exogenous variables that could potentially impact the performance of water companies. Thus, further research efforts are warranted to expand the scope of our analysis and evaluate the influence of additional environmental variables on the productivity change of water companies.

### Supplementary Information

Below is the link to the electronic supplementary material.

Supplementary file1 (DOCX 37 KB)

## Data Availability

Data will be available upon a reasonable request.

## Footnotes

1 A neutral linear programming model is a type of linear programming model where the objective is to optimize a particular objective function while keeping all or some of the decision variables within a specified range or tolerance as the first constraint in Model (3).

2 Unfortunately, in the management reports published by the Superintendencia de Servicios Sanitarios, information about the volume of wastewater treated and its quality is not available.

3 Both quality indices take a value between zero and one, with a value of one suggesting that the water company met all drinking water quality and wastewater treatment standards.
