# Supplementary material for: Assessing the dynamic performance of water companies through the lens of service quality
Source: Environ Sci Pollut Res Int. 2023 Nov 10;30(57):121077–89. doi: 10.1007/s11356-023-30779-z (PMC10697877; doi:10.1007/s11356-023-30779-z)
Supplement: Supplementary file 1 — Supplementary file1 (DOCX 37 KB) [file 11356_2023_30779_MOESM1_ESM.docx]

**APPENDIX**

The mixed-period efficiency ${CE}^{t+1}\left( x^{t},y^{t},b^{t} \right)$ is calculated based on the following linear programming models:

$Max \varphi_{dd}^{t}(x^{t+1},y^{t+1},b^{t+1})=\sum_{l=1}^{k} \mu_{lf}y_{lf}^{t+1}+\sum_{s=1}^{r} \omega_{sf}b_{sf}^{t+1}$ (A1)

$\sum_{i=1}^{m} v_{if}x_{if}^{t+1}=1$

$\sum_{i=1}^{m} v_{if}x_{ij}^{t}-\sum_{l=1}^{k} \mu_{lf}y_{lj}^{t}-\sum_{s=1}^{r} \omega_{sf}b_{sj}^{t}\geq0 j=1,\ldots,n$

$v_{if}\geq0, \mu_{lf}\geq0,\omega_{sf}\geq0$

$Max \beta$ (A2)

$\sum_{i=1}^{m} v_{if}x_{if}^{t+1}=1$

$\sum_{i=1}^{m} v_{if}x_{ij}^{t}-\sum_{l=1}^{k} \mu_{lf}y_{rj}^{t}-\sum_{s=1}^{r} \omega_{sf}b_{sj}^{t}\geq0 j=1,\ldots,n, j\neq f$

$\sum_{l=1}^{k} \mu_{lf}y_{lf}^{t+1}+\sum_{s=1}^{r} \omega_{rf}b_{rf}^{t+1}-\varphi_{dd}^{t}\left( x^{t+1},y^{t+1},b^{t+1} \right)\sum_{i=1}^{m} v_{if}x_{if}^{t+1}=0$

$\mu_{lf}y_{kd}^{t+1}-\beta\geq0 l=1,2,\ldots. k$

$\omega_{sf}b_{sf}^{t+1}-\beta\geq0 s=1,2,\ldots. r$

$v_{if}\geq0, \mu_{lf}\geq0,\omega_{lf}\geq0,\beta\geq0$

The mixed-period efficiency ${CE}^{t+1}\left( x^{t},y^{t},b^{t} \right)$ is calculated based on the linear programming models provided below:

$Max \varphi_{dd}^{t+1}(x^{t},y^{t},b^{t})=\sum_{l=1}^{k} \mu_{lf}y_{lf}^{t}+\sum_{s=1}^{r} \omega_{sf}b_{sf}^{t}$ (A3)

$\sum_{i=1}^{m} v_{if}x_{if}^{t}=1$

$\sum_{i=1}^{m} v_{if}x_{ij}^{t+1}-\sum_{l=1}^{k} \mu_{lf}y_{lj}^{t+1}-\sum_{s=1}^{r} \omega_{sf}b_{sj}^{t+1}\geq0 j=1,\ldots,n$

$v_{if}\geq0, \mu_{lf}\geq0,\omega_{sf}\geq0$

$Max \beta$ (A4)

$\sum_{i=1}^{m} v_{id}x_{id}^{t}=1$

$\sum_{i=1}^{m} v_{if}x_{ij}^{t+1}-\sum_{l=1}^{k} \mu_{lf}y_{lj}^{t+1}-\sum_{s=1}^{r} \omega_{sf}b_{sj}^{t+1}\geq0 j=1,\ldots,n, j\neq f$

$\sum_{l=1}^{k} \mu_{lf}y_{lf}^{t}+\sum_{s=1}^{r} \omega_{sf}b_{sf}^{t}-\varphi_{ff}^{t+1}\left( x^{t},y^{t},b^{t} \right)\sum_{i=1}^{m} v_{if}x_{if}^{t}=0$

$\mu_{lf}y_{lf}^{t}-\beta\geq0 l=1,2,\ldots. k$

$\omega_{sf}b_{sf}^{t}-\beta\geq0 s=1,2,\ldots. r$

$v_{if}\geq0, \mu_{lf}\geq0,\omega_{sf}\geq0,\beta\geq0$

Table A1. Silhouette score

| Number of clusters | Silhouette score |
| --- | --- |
| 2 | 0.2118 |
| 3 | 0.1901 |
| 4 | 0.1968 |
| 5 | 0.2095 |
| 6 | 0.1946 |
| 7 | 0.1498 |
| 8 | 0.1710 |
| 9 | 0.1563 |
| 10 | 0.1373 |
| 11 | 0.1091 |
| 12 | 0.1334 |
| 13 | 0.1169 |
| 14 | 0.1151 |
| 15 | 0.0988 |
| 16 | 0.1107 |
| 17 | 0.1232 |
| 18 | 0.1426 |
| 19 | 0.1082 |
| 20 | 0.0678 |

Figure1. Cluster analysis based on productivity scores of Chilean water companies from 2011 to 2018

Dim 1: Full private water companies

Dim 2: Concessionary water companies

Table A2. Cross efficiency Malmquist productivity index estimations for each Chilean water company evaluated

| Water companies | 2010-11 | 2011-12 | 2012-13 | 2013-14 | 2014-15 | 2015-16 | 2016-17 | 2017-18 | Average |
| --- | --- | --- | --- | --- | --- | --- | --- | --- | --- |
| FPWC1 | 0.970 | 0.982 | 0.950 | 0.980 | 0.997 | 0.971 | 0.967 | 0.986 | 0.975 |
| FPWC2 | 0.962 | 1.023 | 1.035 | 0.992 | 0.972 | 0.974 | 0.941 | 1.031 | 0.991 |
| FPWC3 | 0.958 | 1.017 | 0.994 | 1.074 | 1.002 | 1.046 | 0.984 | 1.011 | 1.011 |
| CWC1 | 1.107 | 1.081 | 1.049 | 1.119 | 1.034 | 1.013 | 1.012 | 1.029 | 1.056 |
| FPWC4 | 0.944 | 0.936 | 0.958 | 0.987 | 1.044 | 1.013 | 0.996 | 1.091 | 0.996 |
| CWC2 | 0.951 | 0.976 | 0.981 | 0.996 | 0.978 | 0.978 | 0.925 | 1.041 | 0.978 |
| CWC3 | 0.947 | 0.974 | 0.927 | 1.053 | 0.997 | 1.001 | 0.984 | 1.013 | 0.987 |
| PWC1 | 0.954 | 0.937 | 0.976 | 1.049 | 0.908 | 0.960 | 0.987 | 1.029 | 0.975 |
| CWC4 | 1.038 | 0.978 | 1.017 | 1.049 | 1.002 | 1.034 | 0.984 | 0.962 | 1.008 |
| CWC5 | 1.038 | 0.988 | 1.017 | 1.049 | 1.002 | 1.034 | 0.983 | 0.961 | 1.009 |
| FPWC5 | 0.973 | 1.066 | 1.025 | 1.144 | 1.001 | 0.923 | 0.972 | 0.995 | 1.013 |
| CWC6 | 1.038 | 1.009 | 1.018 | 1.049 | 0.997 | 1.031 | 0.984 | 0.941 | 1.008 |
| CWC7 | 0.969 | 1.042 | 0.997 | 0.996 | 1.007 | 1.006 | 1.020 | 0.956 | 0.999 |
| FPWC6 | 0.959 | 0.962 | 0.950 | 1.020 | 0.990 | 0.988 | 0.981 | 1.035 | 0.986 |
| CWC8 | 1.002 | 1.011 | 1.077 | 0.970 | 0.881 | 0.971 | 1.058 | 1.026 | 1.000 |
| FPWC7 | 0.977 | 1.130 | 1.171 | 1.105 | 1.020 | 1.182 | 0.989 | 0.913 | 1.061 |
| FPWC8 | 0.953 | 0.960 | 0.935 | 1.000 | 0.943 | 0.981 | 0.947 | 0.959 | 0.960 |
| FPWC9 | 0.980 | 0.928 | 0.992 | 1.020 | 0.972 | 0.995 | 1.025 | 1.056 | 0.996 |
| FPWC10 | 1.019 | 1.012 | 0.984 | 0.992 | 1.003 | 0.947 | 1.000 | 0.987 | 0.993 |
| FPWC11 | 1.004 | 1.015 | 0.978 | 0.893 | 0.837 | 0.911 | 0.854 | 1.041 | 0.942 |
| CWC9 | 0.944 | 1.011 | 0.988 | 0.953 | 1.074 | 1.038 | 0.973 | 0.996 | 0.997 |

Table A3. Cross efficiency change estimations for each Chilean water company evaluated

| Water companies | 2010-11 | 2011-12 | 2012-13 | 2013-14 | 2014-15 | 2015-16 | 2016-17 | 2017-18 |
| --- | --- | --- | --- | --- | --- | --- | --- | --- |
| FPWC1 | 1.016 | 1.016 | 1.035 | 1.009 | 0.947 | 1.016 | 0.945 | 1.131 |
| FPWC2 | 0.980 | 0.979 | 1.078 | 0.963 | 0.910 | 0.985 | 0.972 | 1.223 |
| FPWC3 | 1.010 | 0.947 | 1.038 | 0.985 | 0.993 | 1.030 | 0.954 | 1.085 |
| CWC1 | 1.051 | 1.008 | 0.996 | 1.136 | 1.080 | 1.019 | 0.942 | 1.121 |
| FPWC4 | 0.980 | 0.979 | 1.078 | 0.884 | 1.080 | 1.019 | 0.914 | 1.139 |
| CWC2 | 0.989 | 0.970 | 1.078 | 0.988 | 0.964 | 0.999 | 0.924 | 1.151 |
| CWC3 | 0.997 | 0.864 | 0.992 | 1.112 | 0.993 | 1.030 | 0.954 | 1.099 |
| PWC1 | 1.028 | 0.913 | 1.066 | 1.011 | 0.932 | 1.034 | 0.948 | 1.105 |
| CWC4 | 1.011 | 0.885 | 1.066 | 0.982 | 0.993 | 1.030 | 0.954 | 1.038 |
| CWC5 | 1.011 | 0.885 | 1.066 | 0.982 | 0.993 | 1.030 | 0.954 | 1.017 |
| FPWC5 | 0.956 | 1.031 | 1.029 | 1.213 | 0.989 | 0.982 | 0.991 | 1.102 |
| CWC6 | 1.011 | 0.885 | 1.066 | 0.982 | 0.993 | 1.030 | 0.954 | 0.920 |
| CWC7 | 1.007 | 0.985 | 1.029 | 0.984 | 1.020 | 1.015 | 0.971 | 0.999 |
| FPWC6 | 0.994 | 0.953 | 0.925 | 1.031 | 1.005 | 1.043 | 0.933 | 1.164 |
| CWC8 | 1.090 | 0.838 | 1.096 | 0.956 | 0.958 | 0.935 | 1.155 | 0.933 |
| FPWC7 | 0.999 | 1.001 | 1.150 | 0.915 | 0.872 | 1.107 | 0.965 | 0.924 |
| FPWC8 | 0.997 | 0.997 | 0.973 | 1.004 | 0.953 | 1.044 | 0.932 | 1.071 |
| FPWC9 | 0.990 | 0.881 | 1.068 | 1.025 | 0.957 | 1.050 | 0.980 | 1.105 |
| FPWC10 | 1.085 | 0.995 | 0.949 | 1.027 | 0.974 | 0.991 | 0.978 | 1.027 |
| FPWC11 | 0.999 | 0.931 | 1.037 | 0.982 | 0.768 | 0.981 | 0.933 | 1.255 |
| CWC9 | 1.025 | 0.979 | 1.011 | 0.995 | 1.104 | 1.086 | 0.920 | 1.032 |

Table A4. Cross efficiency technical change estimations for each Chilean water company evaluated

| Water companies | 2010-11 | 2011-12 | 2012-13 | 2013-14 | 2014-15 | 2015-16 | 2016-17 | 2017-18 |
| --- | --- | --- | --- | --- | --- | --- | --- | --- |
| FPWC1 | 0.955 | 0.967 | 0.917 | 0.971 | 1.052 | 0.956 | 1.024 | 0.871 |
| FPWC2 | 0.982 | 1.044 | 0.960 | 1.030 | 1.068 | 0.988 | 0.969 | 0.843 |
| FPWC3 | 0.948 | 1.074 | 0.958 | 1.091 | 1.010 | 1.016 | 1.031 | 0.932 |
| CWC1 | 1.053 | 1.072 | 1.053 | 0.985 | 0.958 | 0.995 | 1.074 | 0.918 |
| FPWC4 | 0.963 | 0.956 | 0.889 | 1.116 | 0.967 | 0.995 | 1.090 | 0.958 |
| CWC2 | 0.961 | 1.006 | 0.910 | 1.008 | 1.015 | 0.979 | 1.001 | 0.905 |
| CWC3 | 0.950 | 1.127 | 0.935 | 0.947 | 1.004 | 0.973 | 1.031 | 0.922 |
| PWC1 | 0.928 | 1.026 | 0.915 | 1.038 | 0.974 | 0.928 | 1.041 | 0.931 |
| CWC4 | 1.026 | 1.105 | 0.954 | 1.069 | 1.009 | 1.004 | 1.031 | 0.927 |
| CWC5 | 1.026 | 1.116 | 0.954 | 1.069 | 1.009 | 1.004 | 1.030 | 0.945 |
| FPWC5 | 1.018 | 1.034 | 0.996 | 0.943 | 1.012 | 0.941 | 0.981 | 0.902 |
| CWC6 | 1.026 | 1.140 | 0.955 | 1.069 | 1.004 | 1.002 | 1.031 | 1.023 |
| CWC7 | 0.962 | 1.058 | 0.968 | 1.012 | 0.988 | 0.992 | 1.051 | 0.956 |
| FPWC6 | 0.965 | 1.009 | 1.027 | 0.989 | 0.984 | 0.948 | 1.051 | 0.889 |
| CWC8 | 0.920 | 1.206 | 0.982 | 1.014 | 0.919 | 1.038 | 0.917 | 1.100 |
| FPWC7 | 0.978 | 1.129 | 1.019 | 1.207 | 1.170 | 1.067 | 1.024 | 0.989 |
| FPWC8 | 0.956 | 0.963 | 0.961 | 0.996 | 0.990 | 0.940 | 1.016 | 0.896 |
| FPWC9 | 0.990 | 1.053 | 0.929 | 0.995 | 1.016 | 0.947 | 1.046 | 0.955 |
| FPWC10 | 0.939 | 1.017 | 1.036 | 0.966 | 1.030 | 0.956 | 1.023 | 0.962 |
| FPWC11 | 1.004 | 1.091 | 0.943 | 0.909 | 1.090 | 0.928 | 0.915 | 0.830 |
| CWC9 | 0.921 | 1.033 | 0.978 | 0.958 | 0.973 | 0.956 | 1.058 | 0.965 |
